# Overcoming Sexual Harassment at University: The Case of the Training Intervention in the Universitat Autònoma de Barcelona

**DOI:** 10.3390/bs15050596

**Published:** 2025-04-29

**Authors:** Olga Serradell, Lidia Puigvert

**Affiliations:** 1 Department of Sociology, Faculty of Political Sciences and Sociology, Autonomous University of Barcelona, 08193 Bellaterra, Spain; 2 Department of Sociology, Faculty of Economics and Business, University of Barcelona, 08034 Barcelona, Spain; lidia.puigvert@ub.edu

**Keywords:** gender-based violence, higher education, isolating gender violence, prevention, bystander intervention, survivors

## Abstract

The social and behavioral sciences have contributed enormously to our understanding of societies and the relationships between individuals within them. Sexual harassment is a universal social problem that is reproduced in different contexts and societies. However, institutions such as universities have made it invisible, contributing to the impunity of harassers and the vulnerability of victims. This has been the case for decades in countries such as Spain. Today, universities are implementing training measures to prevent such behavior. This article analyzes, from a dialogic sociological perspective, one of these interventions in terms of its success and impact on the academic community: the “Training for the Prevention of Gender-Based Violence at the University”, implemented by the Universitat Autònoma de Barcelona (Spain) between 2021 and 2024. The article defines and analyzes its main characteristics: (1) the contents based on scientific evidence; (2) the human commitment of the trainers; and (3) the dialogue with the solidarity network World MeToo Universities. The article concludes that the positive impact of this pioneering intervention in the academic community constitutes a universal and transferable successful action to overcome gender-based violence in universities.

## 1. Introduction

Gender-based violence (hereafter GBV) is a universal social problem that also occurs in the academic environment and includes harassment and sexual violence. In agreement with the Istanbul Convention (Council of Europe, 2011), violence against women is a form of gender-based violence that “shall mean violence that is directed against a woman because she is a woman or that affects women disproportionately” (Council of Europe, 2011, p. 3). Violence against women is reproduced in all social spheres and, as we will see throughout the paper, also in the academic sphere. Sexual harassment is one of the most common forms of GBV in universities and research institutions (Humbert & Strid, 2024). For this reason, in the analysis, although we place emphasis on sexual harassment, we refer to different forms of GBV.

Achieving behavioral changes in this area depends on several factors. One of the most important is the inclusion of scientific evidence as a criterion for all interventions developed (Alzaga et al., 2025; Crespo-López et al., 2025). According to the scientific literature, sexual violence is reproduced in universities from very different countries and cultures, affecting especially women students. A multi-level intersectional analysis of GBV in universities and research institutions in Europe, based on a large cross-cultural survey conducted in 46 research organizations in 15 European countries, found that 62% of respondents experienced at least one form of GBV, with psychological violence (57%) and sexual harassment (31%) being the most common forms (Humbert & Strid, 2024). The study highlights the need to measure GBV to design effective institutional measures.

In their research focused on two South African universities, Treffry-Goatley et al. (2018) highlight that sexual violence in higher education is a significant problem globally, with young women being particularly vulnerable, affecting their mental health, academic performance, and overall well-being. In their article focusing on UK universities, Mudra Rakshasa-Loots (2022) shows GBV as a general problem with serious implications for the lives of younger women students: the results of various national surveys where 48% of women respondents reported experiencing sexual assault, and other independent studies where “52% of university students reported mental illness following their experience of sexual misconduct, and 27% reported a decline in their academic performance (with 20% considering dropping out of university)” (Mudra Rakshasa-Loots, 2022, p. 2). In Spain, the first study on violence against women in a university context (Valls et al., 2016) noted that 62% of students knew or had experienced violent situations. The lack of scientific criteria is particularly serious in preventive GBV interventions, which explains the passive behavior of most members of the university community. They often have difficulties in recognizing violent situations, doubts about how to support victims, or even fear. Other research on support networks in recovery processes for women victims of GBV (Melgar et al., 2021) shows that 40% of the respondents say that they do not act in cases of harassment because they are afraid of retaliation, attacks, or other negative consequences.

The enhancement of academic and professional accomplishments, in addition to scientific output, is closely associated with the existence of secure environments where individuals can engage in scholarly activities without intimidation (Burn, 2009; Edwards-Jauch, 2012; Henry, 2024). Institutions such as universities must include the knowledge provided by scientific evidence to confront the problem of GBV to effectively change behavior and reduce sexual violence in and on their campuses, schools, and departments (classrooms, offices, corridors, libraries, trains, buses). Training the entire university community (students, faculty, staff) means explaining bystander intervention as the most effective mechanism identified by scientific studies to date (Latané & Darley, 1970; Banyard et al., 2007; Banyard, 2011; Burn, 2009; Kuskoff & Parsell, 2024). Henry (2024) highlights the importance of training first responders and bystanders to ensure immediate and effective responses to incidents of violence, and the commitment to annual public reporting of sexual violence data, including the number and outcomes of cases, as an exercise in institutional transparency. The concept of isolating gender violence (hereafter IGV) was created in 2013 by the CREA research group to define “the attacks and retaliation launched against gender violence victims’ supporters so that victims remain isolated” (Vidu et al., 2021, p. 178). Aubert and Flecha (2021) highlighted IGV as a concept that contributes new scientific knowledge, without which it is not possible to understand why some people are afraid to support victims. Therefore, any intervention aimed at preventing GBV and creating safe environments will need to incorporate scientific evidence such as IGV.

Although most universities implement sexual violence prevention training programs, these programs often do not focus on teaching scientific evidence on which interventions are more effective for social impact. This paper presents a successful intervention and its impact on GBV prevention at the university level: the “Training for the Prevention of Gender-Based Violence at the University,” designed, approved, and implemented by the Faculty of Political Sciences and Sociology (hereafter FPSS) of the Universitat Autònoma de Barcelona (hereafter UAB) in Spain (FPSS, 2022b). As we will see below, the information provided by the pioneering training had a positive impact, leading to new and diverse actions for visibility, awareness, and support of victims in different centers of the university.

The origins of this pioneering training intervention can be traced back to the efforts of the CREA (Community of Research on Excellence for All), which was established in 1991 and has been actively engaged in combating GBV in academic institutions (CREA, 2025; Valls et al., 2016). The research’s political implications were significant in Spain and internationally when the Catalan Law 17/2020 was approved on 22 December 2020. This law amended Law 5/2008, which concerns the right of women to eradicate GBV (Catalan Government, 2020, art.3, g). This legislative act incorporated the concept of Second-order Violence, which refers to the reprisals, humiliation, and persecution directed towards individuals providing support to victims of GBV. A comparable approach was adopted by the Basque Country in its Law 1/2022 (BOE, 2022, art. 50. 4). In 2021, the Brazilian Parliament conducted a public hearing on Second-order Violence (Diario Feminista, 2021) at the international level. Consequently, in 2022, a process was initiated in the Brazilian National Congress to draft legislation that incorporates the concept of IGV. This process is currently in progress.

This political impact is associated with the most recent scientific developments in this field. The concept of IGV (Aubert & Flecha, 2021; Vidu et al., 2021) originated from the concept of Second-Order Sexual Harassment (SOSH) (Dziech & Weiner, 1990; Flecha, 2021). In the legislation, this concept was included among other types of violence under the generic term Second-order Violence, as it did not cover only cases of sexual harassment. At that time, scientific advances showed that the isolation of victims was the main consequence and the most relevant element of this type of violence. For this reason, the scientific community coined the term IGV, which is the term we use in this paper and a pivotal component of the training intervention that was the subject of this analysis.

The government program of the FPSS (2021) identified the creation of secure environments for students, teachers, technical, administrative, and service staff as a priority. This objective was fundamental in advancing from the establishment of goals to the implementation of effective interventions that modify behaviors, such as training to prevent GBV. Subsequently, in the training plan approved and implemented in 2022 by the Faculty Board, the IGV emerged as one of the most salient, scientific, evidence-based concepts taught. Furthermore, the political stance of the UAB Rectorate proved to be a key factor in providing support to the FPSS in the formulation of the training plan. The Head of Equality at the University participated in both editions as a speaker and incorporated the definitions of violence included in the Catalan Law 17/2020 into the modification of the general protocol against harassment and GBV of the UAB in December 2022 (UAB, 2022).

## 2. Materials and Methods

We conducted an analysis of the training implemented by the FPSS (2022b) at the UAB in Spain as a pioneering intervention based on international scientific evidence. In the initial stage, we describe the origin of the intervention, elaboration, debate, and approval of the GBV training plan by the Faculty Board in May 2022. In the subsequent stage, we examine the content and evolution of the two training editions, which were conducted in two formats: an in-person session in June 2022 and an online session in December 2022. We assess registration and participation rates and review various documents, including dissemination materials and the communication protocol (FPSS, 2022a, 2022c). In the final stage, an assessment of the social impact of the pioneering training examined here is conducted on other community members in other centers and university units, and the creation of new materials and spaces for debate or the recreation of training content in a different way (UAB, 2022; Monge et al., 2023; Faculty of Science, 2023; School of Engineering, 2024) are examined.

The present analysis will develop a dialogic sociological perspective on the impact of the “Training for the Prevention of Gender-Based Violence at University” (FPSS, 2022b) on the academic UAB community. According to Flecha (2022), the success of interventions to eliminate and prevent violence depends on the dialogue between scientific evidence and the inclusion of the plurality of voices. When both elements are equally prioritized, there is a notable enhancement in the design, particularly in terms of the impact of policies, programs, or training interventions. Dialogic orientation ensures the transferability of action, not only in other countries but also within our own higher education institutions. In alignment with this principle, the analyzed intervention incorporated survivors’ voices and bystander intervention from the outset. Article 27. 1 of the Universal Declaration of Human Rights stipulates that “Everyone has the right to freely participate in the cultural life of the community, to enjoy the arts, and to share in scientific advancement and its benefits” (UNGA, 1948). When actions are derived from this evidence and have already been shown to work to protect and improve the lives of citizens, we can talk about scientific evidence of social impact (Flecha, 2014). It is important to note that access to scientific evidence is the right of all citizens. Moreover, this knowledge is instrumental in preventing the dissemination of false information, hoaxes, and fakes related to the causes and prevention of GBV. For instance, among the most widespread hoaxes are that GBV is more prevalent in stable relationships (Roca, 2020), that “micromachismos” (a term used in Spain to refer to soft violence) are the main cause of GBV (R. Rodríguez, 2020), or even that romantic love triggers gender violence (Yuste et al., 2014).

This paper comprises a literature review that focuses on the primary scientific contributions concerning the prevention and intervention of GBV at the university. The analysis of the information encompasses the material provided in the course by the various trainers, as well as the materials produced during and following the training intervention. Consequently, the study primarily collects information from the literature review and from documents and agreements produced by the faculty that implemented the training and by other centers and bodies of the university. Additionally, information regarding other activities, such as exhibitions or conferences, is collected, based on what was learned in the training or inspired by its contents. The following table lists various representative members of the academic UAB community who developed actions according to scientific evidence learned in the training course (FPSS, 2022b). Some new institutional documents elaborated include a standard to prevent inappropriate behavior between faculty and students. Other contributions include the creation of spaces for dialogue with victims, offering public support, and developing bystander intervention.

Different profiles are identified in terms of position, academic field and category, gender, and age: 1 member of the UAB government (female); 3 deans (2 female, 1 male); 3 faculty members with management positions (2 female, 1 male); and 2 members of the administrative staff (female). Profiles and contributions are described in Table 1.

In a complementary way, we interviewed three key informants (profiles 1, 2, and 3) from Table 1. These individuals provided significant and relevant information. This information allowed us to analyze the actions carried out in their centers. We were able to confirm the positive impact that the training has had. We were also able to identify the potential social impact of the pioneering training intervention for different academic contexts. This is due to the universality and transferability of the intervention analyzed.

The participants were selected based on their leadership roles in their respective centers, namely the Faculty of Biosciences and the School of Engineering. These roles were influenced by the knowledge imparted during the training intervention. Two of the participants held management positions within their respective centers and actively participated in the training. One participant, who currently holds a management position, did not take part in the training but accessed its content through a colleague who was involved. The participants were contacted via email to explain the objective of the study and to provide the questions to be addressed. Following their positive response, a date for the online interview was arranged, with their oral consent recorded at the beginning.

These interviews serve to complement the analysis of the impact of training on the academic community by providing firsthand accounts of significant qualitative value. These accounts offer relevant information that facilitates the identification of the impact of the training under analysis on the behavior of community university members.

To understand the success and impact obtained by the training intervention, it is first necessary to describe the scientific bases on which it is built, considering the already explained institutional context that existed at the UAB at the time the training was approved and implemented.

### 2.1. Science, Commitment, and Dialogue

In 2022, two editions of the training program were conducted: the first in June and the second in December. This intervention is defined as pioneering due to its three main characteristics, which made possible, for the first time, a training space based on science, commitment, and dialogue with victims of GBV. The program’s scientific evidence-based content, the trainers’ expertise in GBV and their commitment to supporting victims, and the inclusion of victim voices through the participation of the solidarity network MeToo University (World MeToo Universities Network, 2025) are three distinguishing characteristics.

#### 2.1.1. The Contents

The training, titled “International Scientific Evidence on Successful Actions in the Prevention and Intervention against Sexual Harassment at the University”, was grounded in empirical findings substantiated by rigorous scientific research and disseminated in leading academic journals in the field.

The first edition was held on 21 June 2022, with the participation of 60 academic members of the FPSS and several members from other UAB centers, including the Faculty of Biosciences and the Faculty of Communication. Other distinguished university members, such as the Head of Administrative Staff in the FPSS and the Faculty of Law, also attended. To ensure the inclusive participation of all faculty members in the training program, the second edition was organized online on 20 December 2022. This edition was disseminated to other UAB centers and staff categories, resulting in a participation of 90 members from diverse academic backgrounds and profiles.

The training was structured into two panels, with each panel comprising five speakers. It is noteworthy that the trainers’ interventions were concise, ranging from 10 to 15 min, with the objective of ensuring the selection of the most salient and pertinent information, maintaining audience engagement, and allocating sufficient time for dialogue, commentary, and inquiries.

The training presented the results of the first study of violence against women on campuses in Spain, published by the most prestigious journal on GBV, *Violence Against Women* (Valls et al., 2016). This pioneering research broke the silence that had prevailed for years in Spanish universities. A salient finding from the survey distributed among a representative sample of 1083 university students was the significant increase in students who were aware of or had experienced GBV during their university years, from 13% to 62%, when specific GBV scenarios were presented, such as psychological violence; sexual aggression; pressure to a have sexual or emotional relationships; non-consensual kissing or touching; discomfort or fear of remarks, looks, emails, phone calls, persecution, or surveillance; dissemination of rumors about one’s sexual life; or remarks with hateful or humiliating sexual connotations, among others. This is explained in the paper:

*First, the researchers asked whether the students knew of any situation of violence against women that had occurred at their university or between people from the university community; only 13% (n = 142) provided an affirmative answer. However, when the students were provided with a particular list of situations (…) 58% (n = 531) of the students who had answered “no” to this question later affirmed that they had experienced or knew someone who had experienced at least one of these situations in the university context (…). Thus, combining the results of these two questions, the percentage of our study participants who had experienced situations of violence against women at their universities or between people belonging to the university community increased from 13–62% (n = 673) when the question provided descriptions of specific situations.*

(Valls et al., 2016, p. 1527)

The audience was also introduced to the initiatives implemented by the leading universities worldwide, including Harvard, UC Berkeley, Wisconsin, and Cambridge, as well as the efficacy of bystander intervention as a key mechanism to prevent GBV. Scientific literature has identified bystander intervention as a strategy to avert sexual violence situations by recognizing risk factors, taking responsibility, deciding to act, and intervening appropriately.

The training provided the academic community with scientific evidence regarding effective intervention strategies for defending and protecting victims. Additionally, it served as a means of addressing the pervasive silence that often enables harassers within various institutions of our society. Thus, as mentioned before, the concept of IGV was a key component of the training, focused on intervention, action, and the adoption of a stance to support and safeguard victims, both individually and institutionally.

The training also included information regarding the ongoing debate in the scientific community concerning the effectiveness and ethical implications of mandatory reporting and mandatory supporting in cases of sexual misconduct in higher education (Holland et al., 2021). Following the identification of unintended consequences resulting from mandatory reporting policies in higher education, the scientific community turned to mandatory support to enhance survivor-centered approaches while maintaining institutional responsibility for addressing sexual misconduct. As pointed out by Holland et al. (2021), mandatory reporting policies often compel survivors to disclose their experiences without consent, leading to increased posttraumatic stress, depression, and anxiety. These policies can discourage survivors from seeking help. On the contrary, mandatory support focuses on listening to survivors, respecting their intentions, and providing trauma-informed support without forcing them to report incidents.

In consideration of the scientific evidence and concurrently with the design and implementation of the training, the FPSS developed a communication protocol that included the support that any victim of sexual harassment would find in our academic community (FPSS, 2022c). To disseminate the documents approved by the Faculty Board in May 2022, other complementary materials were produced, increasing the visibility of the training and victim support actions carried out by the FPSS. These documents and infographics were featured on the FPSS website in a dedicated section entitled “Tell someone!”, along with several news articles highlighting the training intervention and its impact. These articles also served to express support for victims and disseminate scientific evidence. Furthermore, a leaflet was designed for students, with content reviewed by the faculty’s equality committee (FPSS, 2022a). This leaflet included a list of situations of sexual harassment identified by the scientific literature, a communication protocol with information about different ways of making a complaint, people to whom students can go to explain their case, and the statement and institutional support they will find in the case of sexual misconduct. Since that time, at the beginning of each academic year, the FPSS has distributed these leaflets to new students upon their enrollment at the university.

#### 2.1.2. The Trainers

In the formulation of the training plan, several of the most prominent academics in the field were included. Consequently, three of the top ten most cited scholars in the “Gender Violence” category on Google Scholar served as trainers. Notably, one of these trainers led the first, pioneering research on GBV at Spanish universities (Valls, 2005–2008). However, scientific criteria represent a necessary, but not sufficient, requirement for a training program aiming to address GBV within the university environment. A secondary requisite is the establishment of a public stance in support of victims, both from a scientific perspective and on a personal level.

The training program included an institutional positioning of the UAB, exemplified by the notable support of three deans from three different faculties (Psychology, Science, and Political Sciences and Sociology), the director of the Equality Unit, and the university’s highest Equality Representative and General Secretary. In the second edition of the training, the Vice Dean of the Faculty of Biosciences, assistant in the first edition, joined the program as a speaker and explained the interventions developed in her center (Monge et al., 2023). This second edition also included as a speaker the President of AMIT (the Association of Women Researchers and Technologists in Spain) (FPSS, 2022b). At present, it can be affirmed that their support contributed to the impact of this training in other areas.

#### 2.1.3. The Voice of the Victims

One of the trainers was the co-founder of the MeToo University movement, which began in 2013 as a solidarity network for victims and survivors of gender violence on university campuses. This network was established prior to the well-known Hollywood MeToo movement (E. Rodríguez, 2023). Since then, MeToo University members have provided solidarity, listening, and accompaniment to victims of sexual harassment and IGV.

One of the aspects pointed out by the members of the network is to define themselves as survivors, to show others that it is possible to move on despite having suffered this situation (World MeToo Universities Network, 2025; Cañaveras et al., 2024). This represents an important behavioral change with potential consequences for the entire community, as well as for other victims. This means a substantial behavioral shift with the potential to influence the entire community as well as other victims. The subjects express a reluctance to report incidents of violence or sexual harassment, and they are hesitant to disclose their experiences at university in search of support.

The training aimed to make the existence of solidarity networks visible and to give victims a voice. For example, the information provided by the MeToo University speaker highlighted that it is only possible to become a survivor if you have the support and protection of people who stand in solidarity with victims. Today, the movement continues to grow, as does the solidarity of its members and others who bravely support them, such as the professional and quality human journalists who published this press report:

*In January of 2022, twenty-one of us were among the twenty-five women that El Periódico placed on the front page, publicly launching the MeToo University. Among us, there are also four of the first ten most cited female scholars on Gender Violence in Google Scholar. Over these years, and especially since the launching of the MeToo University, the network has been growing. Many and very diverse victims have asked for our support, almost always keeping the confidentiality. Among those who have managed to become survivors, some of them have joined the network.*

(World MeToo Universities Network, 2025: about us/our history)

## 3. Results

After examining the development and content of the training, the most notable elements of the impact generated by this intervention are described and analyzed here.

The social impact of the training program was the result of a co-creation process (Gómez-González et al., 2023) involving diverse members from the UAB. This impact led to the creation of new materials and the expansion of spaces for communication and dialogue with members, representatives, and university units. With diverse interventions, actions, statements, and internal policies, different university centers have recreated the training content of the course. The different ways in which three centers (the Faculty of Science, the Faculty of Biosciences, and the School of Engineering), two libraries (the Science and Technology Library and the Social Sciences Library), and the UAB Training Unit are involved in this process are described below.

### 3.1. Faculty of Science

On 24 January 2023, a significant milestone was attained at the UAB. The “Policy of the Faculty of Science on Situations of Harassment, Sexual Misconduct, and Discrimination” was approved by the Faculty Board (Faculty of Science, 2023). This policy was promoted by the Chair of Equality Policies in the Faculty of Science, the former Dean, and included the scientific concept of IGV. She participated as a speaker in the first edition of the training course and was a key agent in the development of this policy, which contributes to the prevention of GBV at the UAB (see Table 1: 6). Later, the Head of Equality at the university encouraged all faculties and schools at UAB to adapt the document and approve similar policies, a process that is still in progress in several university centers (see Table 1: 8). She also participated as a speaker in the two editions of the training course in 2022 and, as explained later, promoted the recreation of the training contents in two online training sessions aimed at the entire academic community.

They were key actors in the prevention of GBV abroad, as the Catalan Government (2023) developed a document with guidelines to prevent and address situations of GBV in universities, including second-order violence. The policy of the Faculty of Science of the UAB anticipates the protocol of the Catalan government in defining ways to avoid the abuse of power in effective and sexual relationships between the faculty and students.

### 3.2. Faculty of Biosciences and Science and Technology Library

The characteristics of the training course organized by the FPSS led the Vice Dean of the Faculty of Biosciences to design materials and activities that make visible the scientific evidence provided by research and published in the main journals in the field, as well as scientific contributions that have achieved social impact, such as bystander intervention and IGV. As the Vice Dean explained in the interview:

*I’m from the scientific field and I really liked the fact that articles and verified data were presented (…) this is something that influenced me and pushed me to organize the activity that I did afterwards. (…) I also really liked that there were high-quality speakers (…). It’s not useful just to explain points of view, is it? What you need is to know how they do it in other places, how they do prevention, how they approach it, how they talk to teachers, how they detect cases. So, in that sense, I really liked (…) I liked all the speakers, they all spoke in a very scientific and reasoned way.*

(Table 1: profile 3)

Thus, an exhibition and a conference debate with the co-founder of the MeToo University were organized in the Science and Technology Library (hereafter STL) from 6 to 31 March 2023. The Vice Dean and the director of the STL were the organizers, with the collaboration of two deans who participated as trainers in the first edition of the course (see Table 1: 3, 4, 6, 9 profiles). The aim of the exhibition was to promote reflection on the behaviors that constitute GBV and to make visible the tools, resources, and solidarity networks available to victims at the UAB and beyond. These activities showed the effective support of the institution, several deans, and other UAB in the responsibility of the victims, constituting preventive interventions with a very positive impact. On the day of the conference debate with the MeToo University member, she explained very clearly and starkly not only her experience of sexual harassment as a master’s student, but also the lack of support she received from most of her professors and classmates. However, if she had become a survivor at that time, the first victim to win a case at a Spanish university, it was thanks to the support of a professor who knew the scientific evidence of the social impact in the fight against GBV.

The materials and images created are currently open to facilitate other community members to replicate them in their libraries, schools, and faculties. This is what members of other centers have done, such as the Social Science Library (SSL) and the Engineering School (see Table 1: profiles 1 and 5). For example, according to the usage statistics of the UAB Digital Data Repository, since 2023, it has received 1729 views and 1548 downloads. Most of them were from Spain, but also from other countries such as France, the Netherlands, the United States, Germany, Singapore, Mexico, Ireland, Colombia, and China, among others (Monge et al., 2023).

The Vice Dean in charge of these interventions in the Faculty of Biosciences is currently the Chair of Equal Policies in her center. In the interview, she emphasized the need to know exactly what is happening in universities, where it is happening, and what mechanisms we have to intervene and break the false image that GBV does not occur in universities. Her evidence-based knowledge and human commitment are two of the most important factors in ensuring the mandatory support that scientific knowledge identifies as a necessary behavioral change in the academic context.

### 3.3. The UAB Training Based on Scientific Evidence of Social Impact

According to the research (Valls et al., 2016; Melgar et al., 2021), among the most important factors to prevent GBV are: collaborating with and promoting victim support networks; protecting those who support victims, that is, protecting those who protect; ensuring that the policies that govern institutions such as the university are committed to and applied; incorporating the IGV in legislation and protocols; and establishing ways to intervene when GBV occurs or is known to occur at the university. Several of these elements were included in the FPSS (2022b) training, and others have been promoted by key actors at the UAB, thanks to the knowledge provided and discussions held during this pioneering training.

On 1 December 2023 and 15 March 2024, the contents of the FPSS training were recreated as part of the webinar “Prevention of Gender-Based Violence at the University”. Organized by the UAB Training and Equality Units, the course was included in the University’s general training offer. The webinar was divided into two parts, one focusing on the legal framework and the other providing scientific evidence to overcome GBV. This part was entitled “Scientific Evidence of Social Impact for Prevention and Action Against GBV at International Level and in Catalan Universities” and was taught by the Dean of the FPSS (see Table 1: 9).

For the first time, a Spanish university offered training based on scientific evidence of social impact to all its teaching, research, administrative, and service staff. The main agent and promoter of this recreation was the Head of Equality of the whole University (see Table 1: 8), participant and speaker of the first training intervention in 2022 (FPSS, 2022b). With this decision, the Rectorate took a stance to train UAB members according to scientific evidence as a way to overcome GBV, in the same way that medicine cures diseases with evidence-based treatments.

More than 100 people participated in the two webinars, representing a wide range of disciplines and UAB centers. One of these participants was a full professor at the School of Engineering (see Table 1: 1). He became a new key actor in the fight against GBV and IGV at the university, as explained in the following section.

### 3.4. School of Engineering

In the second webinar (15 March 2024), we find the latest impact of pioneer training, which changes the way to intervene and prevent GBV at the university.

A professor at the School of Engineering (see Table 1: 1) participated in the webinar; as he explains, he was attracted by the concreteness of the title focused on prevention and action. During the interview, he explained the impact that the training he received had on him, which led him, in addition to incorporating the content, to include doctoral students in the policy developed, as one of the most vulnerable groups to suffer GBV at the university:

*I was expecting theory, and I saw experience. [The training] affected me a lot because they were real cases. I was embarrassed by the way the institutions treated [the victims], and I found it unbearable. (…) One of the things we did was to include Ph.D. students in this plan, because now they were in limbo.*

(Table 1: profile 1)

He served as Secretary and Vice Director of Academic Affairs in the School of Engineering, one of the largest centers at UAB. In this role, he disseminated information regarding the contents learned to the Vice Director for Gender of the School (see Table 1: 2). Together, they initiated a process in their center to promote and improve the policy on harassment, sexual misconduct, and discrimination, including scientific evidence (School of Engineering, 2024).

The developed policy includes, for example, the definition of IGV and other key elements such as the institutional and public statement to support victims and protect their solidarity network from retaliation. The policy was approved on 4 June 2024, after a very constructive dialogue process based on the scientific evidence presented in the webinar. In addition, the School of Engineering has developed a guide, inspired by the FPSS (2022c) GBV communication protocol, which makes the procedure explicit to be followed in case of non-compliance with the school’s policy on harassment and discrimination.

People from a wide range of academic backgrounds are making progress in preventing GBV in their centers since their actions are based on scientific evidence of social impact that works and have proven to be effective in prevention (Alzaga et al., 2025; Crespo-López et al., 2025).

During the interview, the Vice Director for Gender at the School of Engineering pointed out the importance of positioning those in positions of power at the university, such as professors or political positions, in favor of the victims:

*The cost [of standing up for victims] is very high in sexual violence (…). And unfortunately, you see it when you start your career. One day you enter to do your Ph.D. and one day something happens with I don’t know who and you say, “Oh, this is what happens! And of course, all this is very closed. So maybe we could start working… Because the cost, from my point of view, and maybe I’m wrong, the feeling is that the higher you get, the lower the cost should be. Because when you enter [the university], you put absolutely everything on the line. (…) Maybe we should start with those at the top, who have less at stake.*

(Table 1: profile 2)

The simplicity of some of these actions, such as positioning the institution in favor of victims and their supporters or agreeing on documents such as the one mentioned (School of Engineering, 2024), leads us to have to explain why it has not been done before. Thus, acting to overcome sexual harassment and GBV requires not only knowledge of the scientific evidence, but also a clear political and institutional will to publicize the university’s position in favor of victims. This requires courage and intelligence. However, GBV affects those who publicly demonstrate this support for victims, making them fearful of the reprisals it may have on their professional careers and personal lives. Hence, the importance of each action taken, promoted, or supported by the people highlighted here as key actors (Table 1) is clear, as they have contributed to making the UAB a safer and freer space for everyone today.

## 4. Discussion

From a dialogic sociological perspective, the training analyzed includes the important role of scientific evidence in dialogue with the diverse experiences of victims and bystanders.

Beyond a shift in individual behaviors, bystander intervention (Banyard, 2011) serves as a framework for social transformation and institutional policies that promote proactive engagement in GBV situations. However, even with a comprehensive understanding of the optimal mechanism, further concepts and debates emerge, illuminating the intricate challenges in universities to address GBV and provide effective support to victims. The impact of the training on the academic community may represent a transformation from bystanders to upstanders. The scientific knowledge provided by the training strengthens their personal and institutional commitment to support victims, as they become aware of the multiple options for intervention.

This leads us to develop the following definition of Upstanders: those people who, thanks to the knowledge provided by the scientific evidence of social impact for the prevention of and response to GBV, increase their confidence and commitment to recreating and co-create successful actions that contribute to overcoming the problem in their different contexts and academic fields. In this way, they become key actors and role models for other members of the university community, especially for those who suffer from GBV and IGV.

Improvements in the legal framework already mentioned in this paper contribute greatly to the necessary change in behavior claimed by the MeToo University movement, by including among the forms of violence perpetrated against people who show solidarity with the victims, with the aim of keeping them isolated and without support within the university institution (Joanpere et al., 2022; Aubert & Flecha, 2021). Thus, the concept of IGV gives visibility to the causes of inaction due to fear of reprisals experienced by bystanders (Nazareno et al., 2022). Addressing this fear and adopting a proactive stance is imperative for the global eradication of GBV and sexual harassment in universities.

The impact of the training is closely related to the scientific concept of successful actions (Flecha & Soler, 2014; Soler, 2017), a term that defines those interventions for social inclusion, which are universal and transferable to different socioeconomic and cultural contexts because they are based on scientific evidence of social impact. These interventions have been implemented in various countries worldwide, and the concept of successful actions can be applied in a generic way to different key domains for social inclusion, including education, labor, health, social and political participation, among others. Therefore, it can be concluded that the success of these interventions depends on their scientific evidence base.

In a similar way, the success of the training analyzed here can be attributed to the social impact achieved when informed community agents, who use the scientific knowledge acquired in their schools and faculties in a novel manner, strive to achieve a dual objective: the prevention of GBV and the protection and support of victims.

The training served to empower members to develop different interventions based on scientific evidence. The actions and stances taken by key actors with the support of members of the faculty, the school, or the service collectively contribute to enhancing visibility of GBV and raising awareness among university community members.

Therefore, among the conditions necessary for the effective prevention of gender-based violence in universities, the following stand out: (1) that all members of the university community are aware of and trained in the scientific evidence on social impact; (2) giving victims a voice and maintaining constant dialogue with them; and (3) supporting public, institutional, and personal positioning in favor of victims.

### Limitations and Future Research Directions of the Work

There are two limitations to the work carried out. The first difficulty in cases of GBV among undergraduate and master’s students, i.e., among their peers, is the victims’ refusal to report the harassment through the university’s formal mechanisms, which require them to identify the harasser, risking multiple reprisals. Even taking measures that simply avoid contact, such as changing academic groups, can be reported by the harasser, especially if the harasser has enrolled in the same group as the victim with the aim of perpetuating harassment. The usual result is that the victim drops the subject or even leaves the university. The institutional heads in faculties and schools need greater knowledge and specific ways of implementing preventive measures immediately.

The second limitation is the greater vulnerability of doctoral students. There is an urgent need for these groups to have access to scientific evidence that can effectively prevent GBV at the university. This group of students tends to work in a more isolated way, establishing contact, sometimes almost exclusively, with their thesis supervisor. This can make it difficult to identify a situation of harassment and to report it. In addition, in several Spanish universities, there is the problem that students are not assigned to a center in their academic field but rather belong directly to postgraduate schools with little or no community activity that would allow them to build a network of solidarity or to turn to the heads of the center to which the teaching staff is assigned.

We have seen how the FPSS (2022b) training course has inspired various interventions and institutional documents in different fields. However, it is still relevant to identify the potential social impact of this intervention for more and different academic contexts. Now, other Spanish universities want to replicate the training analyzed in this paper. As a successful, universal, and transferable action, it will be of great scientific interest to analyze the social impact obtained.

Another aspect to continue working on is the protection of those who protect. The concept of IGV places the reproduction of the problem of GBV in the attacks and reprisals against the defenders, with the aim of keeping the victims isolated. One of the most pernicious elements of this is the large number of hoaxes circulating on social networks, in the media, and even among professionals in organizations specializing in violence prevention. Acting from hoaxes instead of scientific evidence has a very negative impact, generating more impunity for harassers and, therefore, more violence.

To combat this misinformation, spaces for public debate and dialogue based on scientific evidence of social impact must be expanded, and these debates should include the first scientists, the survivors, and the upstanders who have taken a stand in favor of victims of GBV at the university.

## 5. Conclusions

This paper is a contribution that provides scientific evidence of social impact to overcome GBV in all universities. Mandatory training is needed in universities worldwide to provide the mandatory support that victims and survivors may need. However, it must be based on scientific evidence to contribute to the eradication of GBV and IGV; otherwise, it will contribute to perpetuating, even increasing, the problem.

The positive impact of the training as a pioneering intervention in the academic community constitutes a universal and transferable successful action for overcoming GBV in universities. This intervention (FPSS, 2022b) has at least three main characteristics that make it unique in the Spanish academic context: Firstly, the knowledge imparted is founded on scientific evidence of social impact. Secondly, dialogue with preeminent scholars on GBV and public supporters is ensured. Thirdly, the voices of survivors are amplified, fostering a more comprehensive understanding of their experiences. This course empowers university members to act effectively in supporting victims of GBV, to overcome the fear of those who support victims, and to achieve prevention.

As we have seen, different participants learned about different ways to be upstanders and contribute to the prevention of GBV at the university. The results described have shown that it is possible and that there are many ways to recreate activities and co-create effective interventions with university members to change attitudes and behaviors that harm victims of GBV and IGV at a university, no matter which field of knowledge they come from.

The main point is to have access to international scientific evidence. From this point on, those who want to effectively end GBV at the university will be able to act on this knowledge from different academic positions and categories. These people will become role models and reinforce the process of moving from being a silent bystander to an upstander who actively supports victims, and from being a victim to becoming a survivor.

## Figures and Tables

**Table 1:** Key actors in GBV prevention activities developed at UAB.

	Position	Center	Contact with the Training	Action Developed	Material
1	Full Professor. Secretary and Vice Director of Academic Affairs.	School of Engineering	Participant in the 4th edition (March 2024).	School of Engineering Policy on Harassment, Sexual Misconduct and Discrimination.	(School of Engineering, 2024).
2	Associate Professor. Vice Director for Gender and Secondary Relations.	School of Engineering	Indirect contact via 1.		
3	Associate Professor. Vice Dean for Mobility, Promotion and Communication.	Faculty of Biosciences	Participant 1st edition (June 2022) and speaker 2nd edition (December 2023).	Exhibition (6–31 March 2023) and MeToo University Conference in the Science and Technology Library, a shared space with three faculties (Biosciences, Sciences and Engineering).	(Monge et al., 2023).
4	Administrative staff. Head of Service.	Science and Technology Library	Indirect contact via 3 and 9.		
5	Administrative staff. Head of Service.	Social Sciences Library	Indirect contact via 4.	Exhibition replicated in the SSL (25 April–19 May 2023).	Using the material created by 1 and 2.
6	Associate professor. Dean and Chair of Equality Policies of the Faculty.	Faculty of Science	Participant and speaker 1st edition (June 2022).	Policy of the Faculty of Science on situations of harassment, sexual misconduct and discrimination.	(Faculty of Science, 2023).
7	Associate Professor. Dean of the Faculty.	Faculty of Science	Indirect contact via 6 and 9.		
8	Associate professor. Head of Equality and General secretary.	Rectorate	Participant and speaker 1st and 2nd editions (June, December 2022).	Promoter of the 3rd and 4th editions of the training as part of the university’s training offer.	(UAB, 2022).
9	Associate professor. Dean of the Faculty.	Faculty of Political Sciences and Sociology	Participant and lecturer in the 4 training editions.	Training promoter and organizer of the 1st pioneer edition as dean.	(FPSS, 2022b).

Own elaboration.

## Data Availability

Data supporting reported results can be found in the UAB Digital Data Repository https://ddd.uab.cat/ (accessed on 27 February 2025).

## Abbreviations

The following abbreviations are used in this manuscript:

FPSS	Faculty of Political Sciences and Sociology
GBV	Gender-based violence
IGV	Isolating Gender Violence
SSL	Social Sciences Library
STL	Sciences and Technology Library
UAB	Universitat Autònoma de Barcelona
